# Accuracy of the 10 μg desmopressin test for differential diagnosis of Cushing syndrome: a systematic review and meta-analysis

**DOI:** 10.3389/fendo.2024.1332120

**Published:** 2024-01-30

**Authors:** Rodrigo Rosa Giampietro, Marcos Vinicius Gama Cabral, Elizandra Gomes Pereira, Marcio Carlos Machado, Lucio Vilar, Vania dos Santos Nunes-Nogueira

**Affiliations:** ^1^Department of Internal Medicine, Sao Paulo State University/UNESP, Medical School, Sao Paulo, Brazil; ^2^Neuroendocrine Unit, Division of Endocrinology and Metabolism, Hospital das Clínicas, University of Sao Paulo Medical School, Sao Paulo, Brazil; ^3^Division of Endocrinology, Hospital das Clínicas, Federal University of Pernambuco, Recife, Brazil

**Keywords:** Cushing syndrome, Cushing disease, pseudo-Cushing syndrome, non-neoplastic hypercortisolism, desmopressin test, systematic review

## Abstract

We evaluated the accuracy of the 10 μg desmopressin test in differentiating Cushing disease (CD) from non-neoplastic hypercortisolism (NNH) and ectopic ACTH syndrome (EAS). A systematic review of studies on diagnostic test accuracy in patients with CD, NNH, or EAS subjected to the desmopressin test obtained from LILACS, PubMed, EMBASE, and CENTRAL databases was performed. Two reviewers independently selected the studies, assessed the risk of bias, and extracted the data. Hierarchical and bivariate models on Stata software were used for meta-analytical summaries. The certainty of evidence was measured using the GRADE (Grading of Recommendations Assessment, Development, and Evaluation Working Group) approach. In total, 14 studies were included: 3 studies on differentiated CD versus NNH and 11 studies on differentiated CD versus EAS. Considering ΔACTH in 8 studies involving 429 patients, the pooled sensitivity for distinguishing CD from EAS was 0.85 (95% confidence interval [CI]: 0.80–0.89, I2 = 17.6%) and specificity was 0.64 (95% CI: 0.49–0.76, I2 = 9.46%). Regarding Δcortisol in 6 studies involving 233 participants, the sensitivity for distinguishing CD from EAS was 0.81 (95% CI: 0.74–0.87, I2 = 7.98%) and specificity was 0.80 (95% CI: 0.61–0.91, I2 = 12.89%). The sensitivity and specificity of the combination of ΔACTH > 35% and Δcortisol > 20% in 5 studies involving 511 participants were 0.88 (95% CI: 0.79–0.93, I2 = 35%) and 0.74 (95% CI: 0.55–0.87, I2 = 27%), respectively. The pooled sensitivity for distinguishing CD from NNH in 3 studies involving 170 participants was 0.88 (95% CI: 0.79–0.93) and the specificity was 0.94 (95% CI: 0.86–0.97). Based on the desmopressin test for differentiating CD from EAS, considering ΔACTH, Δcortisol, or both percent increments, 15%, 19%, or 20% of patients with CD, respectively, would be incorrectly classified as having EAS. For CD versus NNH, 11% of patients with CD would be falsely diagnosed as having NNH, whereas 7% of patients with NNH would be falsely diagnosed as having CD. However, in all hierarchical plots, the prediction intervals were considerably wider than the confidence intervals. This indicates low confidence in the estimated accuracy, and the true accuracy is likely to be different.

**Systematic review registration:**

https://www.crd.york.ac.uk/prospero/display_record.php?RecordID=85634, identifier CRD42018085634; https://www.crd.york.ac.uk/prospero/display_record.php?RecordID=68317, identifier CRD42017068317.

## 1 Introduction

Evaluation of patients with suspected hypercortisolism is one of the most challenging investigations in endocrinology (1). This is due to the intermittent activation of the dynamic hypothalamic–pituitary–adrenal (HPA) axis, which results in clinical and biochemical characteristics that are indistinguishable between neoplastic and non-neoplastic forms of hypercortisolism. Furthermore, even in neoplastic cases, it is often difficult to distinguish between the two main differential diagnoses, namely, endogenous neoplastic hypercortisolism and non-neoplastic hypercortisolism (NNH) (1).

In adults, the most frequent etiology of endogenous neoplastic hypercortisolism is Cushing disease (CD), accounting for approximately 70% of Cushing syndrome (CS) cases (2). CD is caused by increased production of adrenocorticotropic hormone (ACTH) due to pituitary adenoma. It has an incidence and prevalence of 2–3 cases per 1,000,000 inhabitants/year and 40 cases per 1,000,000 inhabitants, respectively (3). The principal differential diagnosis of CD is endogenous neoplastic hypercortisolism secondary to ectopic production of ACTH (ectopic ACTH syndrome [EAS]), which accounts for 10%–20% of the causes of ACTH-dependent CS (4).

Patients with NNH (previously known as pseudo-Cushing syndrome) have been recognized for over 50 years (5). These individuals show mild-to-moderate ACTH-dependent hypercortisolism due to alcohol use disorder, neuropsychiatric disorders, chronic kidney disease, or poorly controlled diabetes mellitus (5–9).

When the prevalence of one of the conditions that characterize NNH increases, many patients with endogenous neoplastic hypercortisolism may not develop the most specific signs and symptoms associated with this hormonal disorder (e.g., easy bruising, capillary fragility, proximal weakness, and reddish-purple striae). Thus, there is an urgent need to distinguish these two clinical conditions. Additionally, as pituitary microadenomas may be present in 9.3% (range, 1.5%–26.7%) of pituitary incidentalomas in the general population (10) and in up to 38% of patients with EAS (11), the differential diagnosis between CD and EAS has been recommended (7, 12), especially when a lesion with a size of <6 mm is observed on pituitary magnetic resonance imaging (MRI).

Regarding differential diagnosis between CD and EAS, the gold standard examination is bilateral and simultaneous petrosal sinus sampling (BIPSS). This method exhibits a diagnostic accuracy of 90%–98% (13–15). However, BIPSS is invasive and should be performed by highly qualified professionals (7); these factors have limited its widespread use. Therefore, some dynamic tests have been developed for the differential diagnosis of endogenous CS.

The corticotropin-releasing hormone (CRH) test, dexamethasone-suppressed CRH stimulation test (DEX-CRH test), and desmopressin stimulation test have been widely used to distinguish neoplastic hypercortisolism from NNH as well as perform differential diagnosis between CD and EAS (16–18). However, the current lack of availability of CRH for diagnostic purposes, even in countries where it was previously used, has led to increased use of the desmopressin stimulation test to examine HPA axis function (1, 19).

Although these dynamic tests have been studied in detail in CS, no evidence synthesis with meta-analysis has focused on the desmopressin test. Thus, we aimed to evaluate the diagnostic accuracy of the desmopressin test at an intravenous dose of 10 μg to distinguish neoplastic hypercortisolism from NNH and perform differential diagnosis between CD and EAS.

## 2 Methods

A systematic review was conducted according to the Cochrane Handbook for Systematic Reviews of Diagnostic Test Accuracy (20, 21), and the results were reported according to the PRISMA-diagnostic test accuracy (DTA) studies criteria (22). The protocol was registered in the International Prospective Registry of Systematic Reviews (IDs : CRD42018085634 and CRD42017068317).

### 2.1 Eligibility criteria

We included the DTA studies that followed the PIRO structure described below.

#### 2.1.1 Population (P)

Patients with clinical suspicion of endogenous CS who underwent at least two different screening tests for hypercortisolism: 24-h urinary free cortisol (UFC), late night salivary cortisol, no suppression of serum cortisol after the administration of 1 mg dexamethasone overnight, or no suppression after the administration of 2 mg dexamethasone for 48 h.

#### 2.1.2 Test index (I)

We considered desmopressin administered at an intravenous dose of 10 µg as the test index. Serum cortisol and plasma ACTH levels were measured at 15 and 0 min before and 15, 30, 45, 60, and 90 min after desmopressin administration.

#### 2.1.3 Reference test (R)

Patients diagnosed with an ACTH-secreting pituitary adenoma during pathologic analysis after pituitary surgery were considered to have CD. Patients who did not undergo any surgery were considered to have CD if their plasma ACTH level was >10 pg/mL and if they met one of the following criteria: BIPSS with a central-to-peripheral ratio of plasma ACTH level of ≥2.0 pg/mL before or ≥3.0 pg/mL after CRH test or desmopressin administration, and the presence of a pituitary adenoma measuring >6 mm on MRI in a patient with concordant results suggestive of CD based on the high-dose dexamethasone suppression test (HDDST) and CRH or desmopressin stimulation tests (7).

EAS was diagnosed through immunohistochemical analysis of tumor tissues. In the absence of surgery or immunohistochemistry negative for ACTH expression, which can be noted in up to 30% of EAS cases (11, 23, 24), the absence of central gradient of ACTH at BIPSS (25) or improvement in hypercortisolism after surgery was considered.

A diagnosis of NNH was made in patients with major depression, obsessive–compulsive disorder, anxiety disorder, chronic alcoholism, or severe obesity as well as in those who exhibited hypercortisolism resolution at follow-up after the control of NNH-associated disease (8, 9, 26).

#### 2.1.4 Outcomes (O)

Using a 2 × 2 contingency table, the performance of the desmopressin test was compared with that of the reference test, in which true-positive, false-positive, false-negative, and true-negative cases were determined for CD diagnosis. Based on these data, the accuracy of the index test (sensitivity, specificity, positive likelihood ratio [LR+], and negative likelihood ratio [LR−]) was calculated.

#### 2.1.5 Exclusion criteria

Studies involving patients who were diagnosed with CD without presenting the abovementioned confirmatory criteria were excluded. Moreover, studies involving patients with NNH who did not undergo outpatient follow-up for evaluating hypercortisolism after the resolution of NNH-associated disease were excluded. Studies including patients who were diagnosed with CD or EAS without presenting the abovementioned confirmatory criteria were also excluded.

### 2.2 Search strategies

Four general search strategies were implemented for the EMBASE (1980-10/10/2017), PubMed (1966-10/10/2017), LILACS (1982-10/10/2017), and CENTRAL (Cochrane Collaboration Controlled Trials Registry-10/10/2017) electronic databases (**Supplementary File**). All databases were searched for the second time on September 25, 2023. The index terms “Cushing disease” and “desmopressin” were used to establish each search strategy with no language or year restrictions. EndNote X9 citation management software was used to download the references and remove duplicate entries. For initial screening of abstracts and titles, the free web application Rayyan QCRI was used (27).

### 2.3 Study selection

Four reviewers independently and in pairs (RRG, MVGC, EGP, and VSN-N) selected titles and abstracts from the reference articles identified through bibliographic search. After selecting potentially eligible studies, the full-text was reviewed. The studies were evaluated for conformance to the proposed PIRO structure. In case of disagreements during the selection process, a consensus was achieved through discussion. The reasons for the exclusion of each study were justified.

### 2.4 Data extraction and management

Two reviewers extracted data regarding study characteristics and the corresponding participant-related information for each study. For each comparison between index and reference tests, data regarding the number of true-positive, true-negative, false-positive, and false-negative cases were extracted in the form of a 2 × 2 table.

### 2.5 Risk of bias and applicability

The risk of bias associated with the included studies was evaluated using the Quality Assessment of Diagnostic Accuracy Studies tool (28).

### 2.6 Unit of analysis

The unit of analysis was the aggregate data extracted from the journal publications.

### 2.7 Synthesis of results (meta-analysis)

For each study, a 2 × 2 contingency table was constructed. Sensitivity, specificity, and LRs were calculated. When the primary study had a value of 0 in a cell of the 2 × 2 table, the value of 1 was added to facilitate calculations (29); this was observed in two of the included studies.

We performed meta-analyses using hierarchical and bivariate models, which account for variability in intrastudy accuracy as well as interstudy variations in test performance with the inclusion of random effects (30). Based on the results of heterogeneity investigations, the bivariate model was used to estimate summary sensitivity and specificity (summary points), and the hierarchical summary receiver operating characteristic (HSROC) model was applied to construct summary ROC curves.

Stata Statistical Software V.18 (StataCorp LLC), with metadta and metandi commands, was used for analyses.

### 2.8 Assessment of heterogeneity

Forest and HSROC plots were visually assessed for heterogeneity. If data allowed, we evaluated the sources of heterogeneity through subgroup analyses. Meta-regression could not be performed because of the limited number of studies available. Variability away from the summary ROC curve is likely to represent greater heterogeneity than variation along the summary ROC curve, which might correspond to simple threshold effects. If the number of studies included was adequate, we would assess the following potential heterogeneity sources: patient characteristics, test methods, and study design. A separate SROC curve would be fitted for each subgroup, and the results would be compared graphically across subgroups (30).

### 2.9 Sensitivity analyses

If the number of studies selected was adequate, we assessed the robustness of our results by conducting sensitivity analysis according to the threshold of ACTH level and cortisol percent increment after the desmopressin test.

### 2.10 Grading of the quality of evidence

For each outcome, the findings were summarized in a tabulated format to determine the effectiveness of the index test. The certainty of evidence was measured using the GRADE (Grading of Recommendations Assessment, Development, and Evaluation Working Group) approach (31, 32).

## 3 Results

### 3.1 Study selection

**Figure 1:**
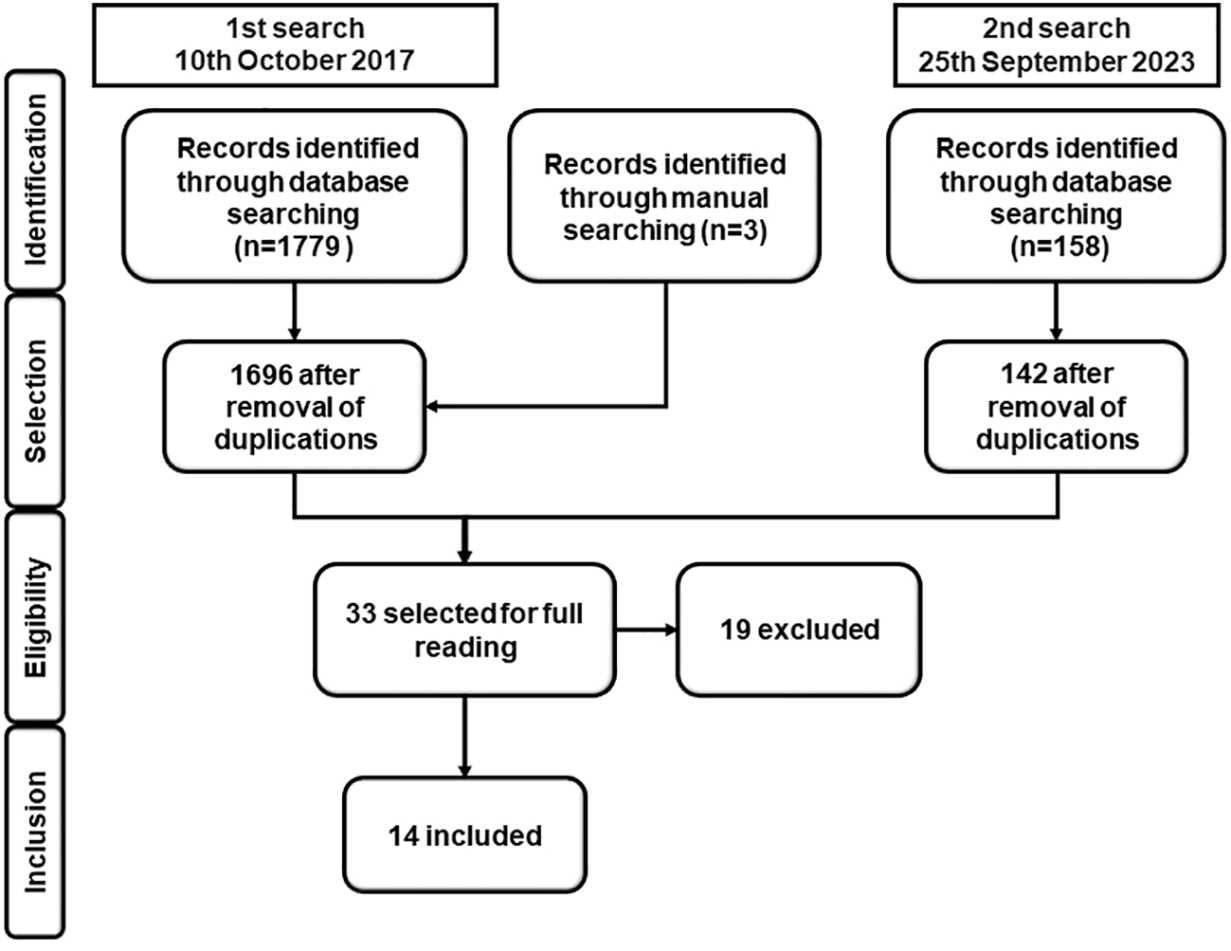
Flowchart of the identification of eligible studies.

The search strategies yielded 1,940 references. After removing duplicates, 1,838 studies remained (**Figure 1**). Thirty-three studies potentially eligible for inclusion in the full-text review were selected. However, of these, 19 studies were excluded for the following reasons. One study was a narrative review article (6), and seven studies did not use the desmopressin test as the index test (33–39). In another study, the authors used the desmopressin test to distinguish patients with CD from those with a clinical and laboratory suspicion of CS. In the same study, although most patients suspected of CS had undergone at least one positive screening test for hypercortisolism, they were not classified as carriers of NNH (40). Two studies compared the results of desmopressin test in patients with CD and those with depression; however, the patients with depression showed no clinical or laboratory features of CS (41, 42). Three studies involved patients who were previously included in a published series (43–45). Another study (46) had no patients with EAS in their series. Salgado et al. (47) evaluated the desmopressin test results in patients with EAS, and no patient with CD was included in their series. Sakai et al. and Suda et al. conducted the desmopressin test with 5 and 4 µg of desmopressin, respectively (48, 49). In another study, the criteria used for distinguishing CD from NNH were not described (50).

### 3.2 Study characteristics

According to our eligibility criteria, we included the following 14 studies: 3 studies distinguishing CD from NNH (51–53) and 11 studies distinguishing CD from EAS (54–64). These studies included 979 participants (782 with CD, 79 with NNH, and 118 with EAS). Five of the included studies also involved a group of healthy individuals who underwent desmopressin tests. **Tables 1**, **2** present the descriptive data of the included studies on differential diagnosis of CD versus EAS and CD versus NNH respectively.

**Table 1:** Characteristics of the studies included in relation to the “PIRO” and the contingency table for the accuracy of the 10 µg desmopressin test to distinguish Cushing disease (CD) from ectopic ACTH syndrome (EAS).

Study/country	Study design	Patients (n)	Assays	Reference test description	Cutoff descriptions	TP (n) CD/EAS	FP (n) CD/EAS	FN (n) CD/EAS	TN (n) CD/EAS
Ferrante, 2022 (54) Italy	Retrospective comparative cohort (2000−2017)	CD: 148 EAS: 26*	Cortisol: RIA/ECLIA ACTH: IRMA/ECLIA	CD and EAS: ACTH > 20 ng/L, pituitary MRI, BIPSS, histopathology	ΔACTH > 30% plus Δcortisol > 20%	104	11	44	11
Frete, 2020 (55) France	Retrospective comparative cohort	CD: 167 EAS: 27	IMMULITE and RIA	CD: histopathology and remission following trans-sphenoidal surgery (n =154 patients),BIPSS EAS: pathology in 25/27 cases	ΔACTH > 33% plus Δcortisol > 18% (ROC-based)	139	5	28	22
Qiao, 2021 (56) China	Retrospective comparative cohort	CD: 92 EAS: 16	–	CD: histopathology, biochemical remission after Gamma Knife treatment for pituitary lesions EAS: BIPSS	ΔACTH > 35% or Δcortisol > 20% or ΔACTH > 35% plus Δcortisol > 20%	77 78 85	2 2 5	15 14 7	14 14 11
Colombo, 1997** (58) Italy	Retrospective comparative cohort	CD: 17 EAS: 1	Cortisol: RIA ACTH: IRMA	CD: histopathology EAS: BIPSS	ΔACTH > 50% plus Δcortisol > 20%	16	0	1	1
Malerbi, 1993** (59) Brazil	Retrospective comparative cohort	CD: 16 EAS: 1	Cortisol: RIA ACTH: RIA/IRMA	CD: biochemical remission, histology, or BIPSS	Δcortisol > 40%–44%	12	0	1	1
Newell Price, 1997 (61) England	Retrospective comparative cohort	CD: 17 EAS: 5***	Cortisol and ACTH: RIA	CD: histopathology (n = 24) BIPSS (n = 1)	ΔACTH > 35% or Δcortisol > 20% or ΔACTH > 35% plus Δcortisol > 20%	12 14 12	3 1 1	5 3 5	1 3 3
Terzolo, 2001 (62) Italy	Retrospective comparative cohort	CD: 34 EAS: 9	Cortisol: RIA ACTH: IRMA	CD: histopathology (n = 29); After surgery (n = 5) EAS: After surgery (n = 8); occult (n = 1) (BIPSS)	ΔACTH > 35% plus D > 4.5 pmol/L or ΔACTH > 50% plus D > 4.5 pmol/L or ΔACTH > 35% or ΔACTH > 50% or Δcortisol > 20% plus D > 193 nmol/L	17 16 17 16 15	2 2 3 3 3	2 3 2 3 4	3 3 2 2 2
Tsagarakis, 2002 (63) Greece	Retrospective comparative cohort	CD: 26 EAS: 5	Cortisol: RIA ACTH: IRMA	CD: histopathology (n = 14); biochemical remission (n = 6); BIPSS (n = 6) EAS: after surgery (histology + IHC)	ΔACTH > 50% or Δcortisol > 20%	21 19	3 3	5 7	2 2
Vilar, 2008 (64) Brazil	Retrospective comparative cohort	CD: 46 EAS: 7 Adrenal CS: 21 CD: 21 EAS: 4	Cortisol: ICMA ACTH: IRMA/ICMA	CD: ACTH immunostaining of pituitary adenoma and/or reversal of hypercortisolism after surgery EAS: ACTH immunoreactivity of neoplastic tissue (n = 7) or BIPSS	ΔACTH ≥ 35% or ΔACTH ≥ 50% or Δcortisol ≥ 20% or Δcortisol ≥ 50%	18 16 16 10	1 0 1 1	3 5 5 11	3 4 3 3
Marova, 2008 (60)	Retrospective comparative cohort	CD: 21 EAS: 11	ACTH: CIS-bio-International Cortisol: ECI automatic analyzer	CD: MRI with ACTH antibodies EAS: CT	ΔACTH > 30% or Δcortisol > 30%	16	1	5	10
Barbot, 2016 (57) Italy	Retrospective comparative cohort (2003–2013)	CD: 149 EAS: 21	Cortisol: RIA/ECLIA ACTH: IRMA/ECLIA	CD: biochemical remission after surgery, histology and/or temporary hypoadrenalism EAS: histopathology (n = 20); BIPSS (n = 1)	ΔACTH > 32.3%	124	8	25	13

ACTH, adrenocorticotropic hormone; AI, adrenal insufficiency; BIPSS, bilateral and simultaneous petrosal sinus sampling; CD, Cushing disease; CS, Cushing syndrome; CT, computed tomography, D, delta; EAS, ectopic ACTH syndrome; ECLIA, electrochemiluminescence immunoassay; FN, false-negative; FP, false-positive; ICMA, immunochemiluminometric assay; IHC, immunohistochemistry; IRMA, immunoradiometric assay; MRI, magnetic resonance imaging; RIA, radioimmunoassay; ROC, receiver operating characteristic; TN, true-negative; TP, true-positive.

*14 patients were also presented in the Barbot study, **a few patients did not show ACTH levels of >10 pg/mL during CS diagnosis; ***only four EAS cases had ACTH and cortisol increments evaluated; –, no information provided.

**Table 2:** Characteristics of the studies included in relation to the “PIRO” and the contingency table for the accuracy of the10 µg desmopressin test to differentiate Cushing disease from non-neoplastic hypercortisolism.

Study/country	Study design	Patients (n)	Assays	UFC 24 h (nmol/24 h)	Reference test description	Cutoff for CD	TP (n)	FP (n)	FN (n)	TN (n)	Follow-up
Tirabassi, 2010 (52) Italy	Retrospective comparative cohort	CD: 52 NNH: 28 CG: 31	I	CD: 778 (484.2–1,545), NNH: 526.9 (95% CI: 461.3–620.7)	CD: pituitary surgery and/or postoperative clinical and biochemical resolution of hypercortisolism	Basal cortisol > 331 nmol/L and ACTH > 4 pmol/L	20*	2	3*	26	3 years
Giraldi, 2007* (53) Italy	Retrospective comparative cohort	CD: 29 NNH: 23 CS: 32	I UFC: RIA	CD: 707.7 (SD: 87.0) NNH: 279.7 (± 41.15)	CD: pathology on trans-sphenoidal surgery specimen	ΔACTH > 6 pmol/L	15*	2	4*	19	1 and 3 years for NNH
Moro, 2000* (51) Italy	Retrospective comparative cohort	CD: 76 NNH: 30 CG: 31	ACTH: I UFC and cortisol: RIA	CD: 818 (± 122.14) NNH: 321.7 (± 27.81)	CD: pituitary surgery and/or postoperative clinical and biochemical resolution of hypercortisolism	ΔACTH > 6 pmol/L	18*	1	2*	29	2 years for NNH

ACTH, adrenocorticotropic hormone; CD, Cushing disease; CG, healthy control group; CI, confidence interval; FN, false-negative; FP, false-positive; I, immunometric; NNH, non-neoplastic hypercortisolism; RIA, radioimmunoassay; TN, true-negative; TP, true-positive; UFC, urinary free cortisol.

*Patients with CD and mildly elevated UFC 24 h.

All studies conducted intravenous desmopressin tests, wherein a slow bolus of 10 μg desmopressin was injected into the antecubital vein of patients who had fasted overnight. This was followed by the measurement of plasma ACTH and serum cortisol levels at 15 and 0 min before and 10, 20, 30, 45, 60, 90, and 120 min after desmopressin administration. Only Terzolo et al. (62) excluded the 120-min time point from their protocol. Barbot et al. used the following time points: 15 and 0 min before and 15, 30, 45, 60, 90, and 120 min after desmopressin administration (57). The baseline ACTH and cortisol levels were expressed as the means of the respective measurements taken between 15 and 0 min before desmopressin administration. The absolute increase in plasma ACTH levels after desmopressin administration was defined as the difference between the value at 0 min and the highest value attained within 30 min (ΔACTH).

Regarding differential diagnosis of CD versus NNH, the patients in the included studies were suspected of having endogenous CS, and most of them had mild hypercortisolism. A similar definition of mild hypercortisolism was used in all included studies. Tirabassi et al. defined mild hypercortisolism as a 24-h UFC level of <771 nmol/day (~2 times of the upper limit of normal range [ULNR]), whereas Moro et al. (51) and Giraldi et al. (53) defined it as a 24 - h UFC level of <690 nmol/day (~3 times of the ULNR). Regarding the criteria for differentiating CD from NNH, a study defined CD as ΔACTH of >4 pmol/L along with a baseline serum cortisol threshold of >331 nmol/L (52), whereas other studies defined CD as ΔACTH of >6 pmol/L (51, 53).

To distinguish CD from EAS, eight studies calculated sensitivities and specificities based on ΔACTH percent increment, six studies calculated these values based on Δcortisol percent increment, whereas five studies calculated the sensitivity and specificity based on both, Δcortisol and ΔACTH percent increment. For CD diagnosis, the most used criteria were ΔACTH of >35% and Δcortisol of >20% (**Table 1**). Most criteria used in these studies were prespecified by the authors.

### 3.3 Risk of bias

**Figure 2:**
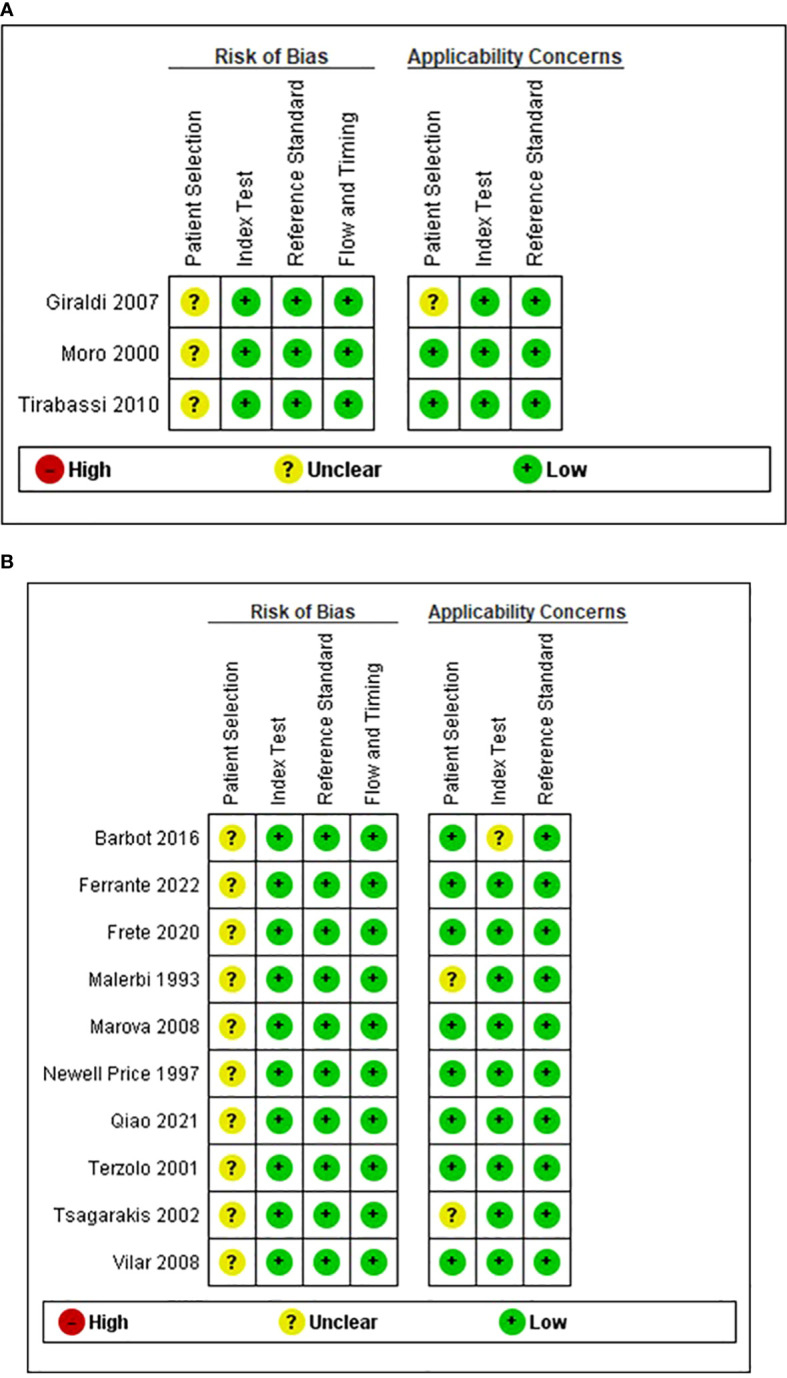
Risk of bias and applicability concerns: authors’ judgment on each domain for all included studies. **(A)** Desmopressin test to distinguish Cushing disease from non-neoplastic hypercortisolism. **(B)** Desmopressin test to distinguish Cushing disease from ectopic ACTH syndrome.

**Figure 2** summarizes the overall methodological quality of all included studies. These studies retrospectively evaluated CS patient series that required differential diagnosis of CD versus EAS or CD versus NNH. However, these studies did not report whether participant recruitment was performed randomly or consecutively. Therefore, all included studies were considered as having an unclear risk of bias for patient selection. Barbot et al. (57) did not prespecify the threshold used; therefore, we considered that their study had an unclear applicability concern for the index test. We revealed that other studies and domains had a low risk of bias and applicability concern.

### 3.4 Data syntheses–meta-analyses

#### 3.4.1 Distinguishing CD from EAS using the desmopressin test (10 ug)

**Figure 3:**
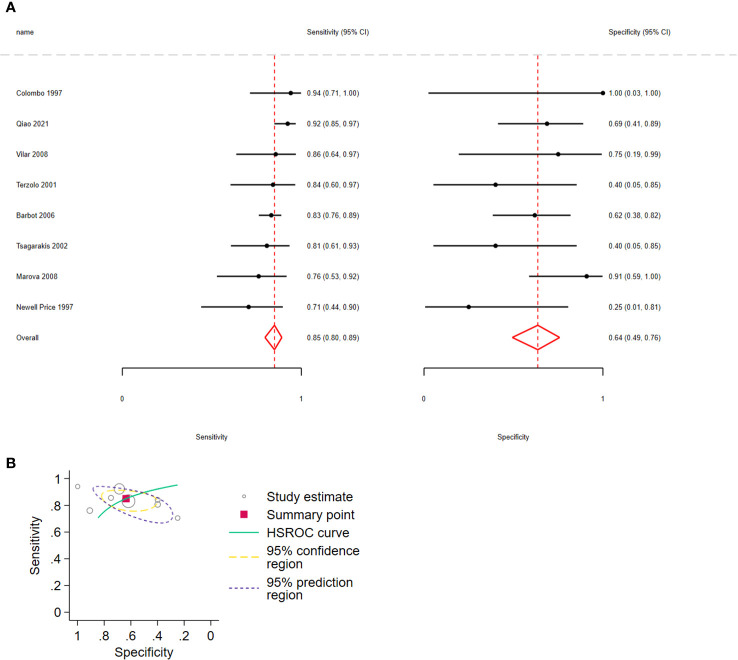
**(A)** Forest plot depicting the sensitivity and specificity considering ACTH percent increment after 10 µg desmopressin test to distinguish Cushing disease from ectopic ACTH syndrome. The figure indicates the estimated sensitivity and specificity of the study (black circle) and its 95% confidence interval (black horizontal line). **(B)** Summary ROC plots from Stata after fitting the hierarchical model to ACTH percent increment. The circles represent the estimates of individual primary studies, and square indicates the summary points of sensitivity and specificity. HSROC curve is plotted as a curvilinear line passing through summary point. The 95% confidence interval and 95% prediction interval are also provided. HSROC, hierarchical summary receiver operating characteristic.

Considering ΔACTH, the pooled sensitivity for distinguishing CD from EAS was 0.85 (95% CI: 0.80–0.89, I2 = 17.6%) and pooled specificity was 0.64 (95% CI: 0.49–0.76, I2 = 9.46%); 8 studies, 429 patients, low certainty of evidence (**Figure 3A**; **Table 3**). The LR+ was 2.33 (95% CI: 1.58–3.45) and LR− was 0.24 (95% CI: 0.15–0.36).

**Table 3:** Summary of the proposed “PIRO” and the pooled sensitivity and specificity results of the accuracy of the 10 µg desmopressin test to distinguish Cushing disease (CD) from ectopic ACTH syndrome (EAS) and certainty of evidence according to the GRADE (Grading of Recommendations Assessment, Development, and Evaluation) approach.

What is the accuracy of the 10 µg desmopressin test in distinguishing Cushing disease (CD) from ectopic adrenocorticotropic hormone (ACTH) syndrome (EAS)?					
Population: Patients with ACTH-dependent Cushing syndrome (CS)					
Prior testing: None					
Settings: Ambulatory health care settings					
Index test: Desmopressin (10 µg, intravenously) with serum cortisol and plasma ACTH levels					
Reference standard: ACTH-producing hormone in the pathologic analysis or simultaneous bilateral inferior petrosal sinus sampling (BIPSS)					
Importance: Accurate and rapid diagnosis allows appropriate and rapid treatment for CD with no need for additional investigations for ectopic ACTH syndrome					
Studies: Retrospective cohort studies, 11 studies involving 809 individuals					
Quality concerns: Few studies and sparse data					
Test/subgroup	Accuracy summary (95% CI)	Number of participants	CD prevalence	Implications	Quality comments/certainty of evidence according to the GRADE approach
Cutoff: ACTH increment >35% (5 studies) and >50% (3 studies)	Sensitivity: 85% (80%–89%) Specificity: 64% (49%–76%)	429 (8 studies)	362/429 = 84%	With a prevalence of 84%, 84/100 patients with ACTH-dependent Cushing syndrome will have CD. Of them, 13 will be missed by the desmopressin test (16% of 84). Patients with CD but falsely diagnosed with EAS will be subjected to an extensive investigation to determine the source of ectopic ACTH production. Of the 16 patients with EAS, 6 will be misdiagnosed as having CD and may be unnecessarily referred for MRI and sometimes for IPSS	All studies were evaluated as having an unclear risk of bias for patient selection; the prediction interval was considerably wider than the confidence interval. Low certainty of evidence
Cutoff: Cortisol increment >30%; >20% (4 studies); >30%	Sensitivity 81% (74%–87%) Specificity: 80% (61%–91%)	233 (6 studies)	191/233 = 82%	With a prevalence of 82%, 82/100 patients with ACTH-dependent Cushing syndrome will have CD. Of them, 16 will be missed by the desmopressin test (19% of 82). Patients with CD but falsely diagnosed with EAS will be subjected to an extensive investigation to determine the source of ectopic ACTH production. Of the 18/100 patients with EAS, four may be unnecessarily referred for MRI and sometimes for IPSS	All studies were evaluated as having an unclear risk of bias for patient selection; the prediction interval was considerably wider than the confidence interval. Low certainty of evidence
Cutoff: Cortisol increment >20% plus ΔACTH >35%, 5 studies	Sensitivity 80% (73%–86%) specificity 74% (55%–87%)	511 (6 studies)	441/511 = 86%	With a prevalence of 86%, 86/100 patients with ACTH-dependent Cushing syndrome will have CD. Of them, 17 will be missed by the desmopressin test (20% of 86). Patients with CD but falsely diagnosed with EAS will be subjected to an extensive investigation to determine the source of ectopic ACTH production. Of the 18 patients without CD, 5 may be unnecessarily referred for MRI and sometimes for IPSS	All studies were evaluated as having an unclear risk of bias for patient selection; the prediction interval was considerably wider than the confidence interval. Low numbers of patients per study. Very low certainty of evidence

**Figure 4:**
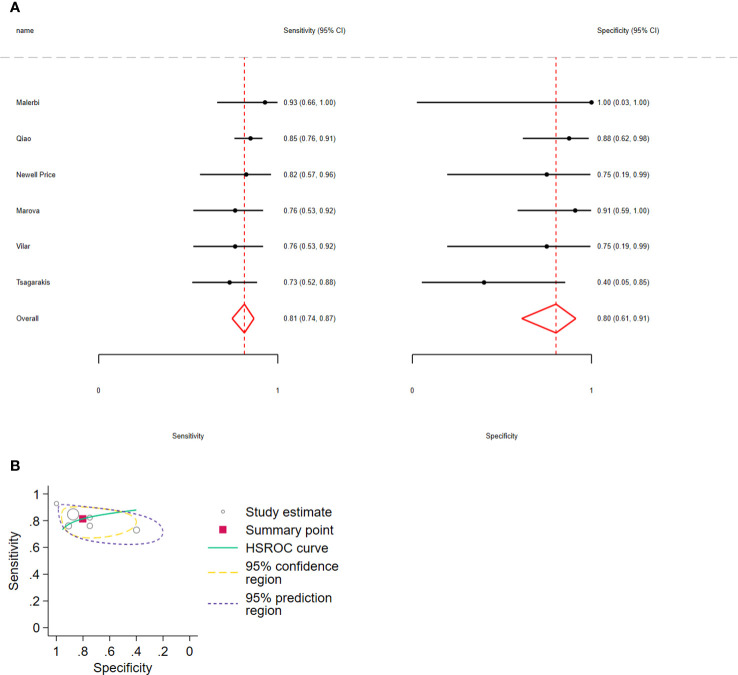
**(A)** Forest plot depicting the sensitivity and specificity considering the cortisol percent increment after the 10 µg desmopressin test to distinguish Cushing disease from ectopic ACTH syndrome. Estimated study sensitivity and specificity (black circle); 95% confidence interval (black horizontal line). **(B)** Summary ROC plots from Stata after fitting the hierarchical model to cortisol percent increment. Circles represent the estimates of individual primary studies, and squares indicate the summary points of sensitivity and specificity. HSROC curve is plotted as a curvilinear line passing through the summary point. The 95% confidence interval and 95% prediction interval are also provided. HSROC, hierarchical summary receiver operating characteristic.

Regarding Δcortisol, the pooled sensitivity for distinguishing CD from EAS was 0.81 (95% CI: 0.74–0.87, I2 = 7.98%) and pooled specificity was 0.80 (95% CI: 0.61–0.91, I2 = 12.89%); 6 studies, 233 participants, low certainty of evidence (**Figure 4A**; **Table 3**). The LR+ was 4.1 (95% CI: 1.9–8.94) and LR− was 0.23 (95% CI: 0.15–0.35).

**Figure 5:**
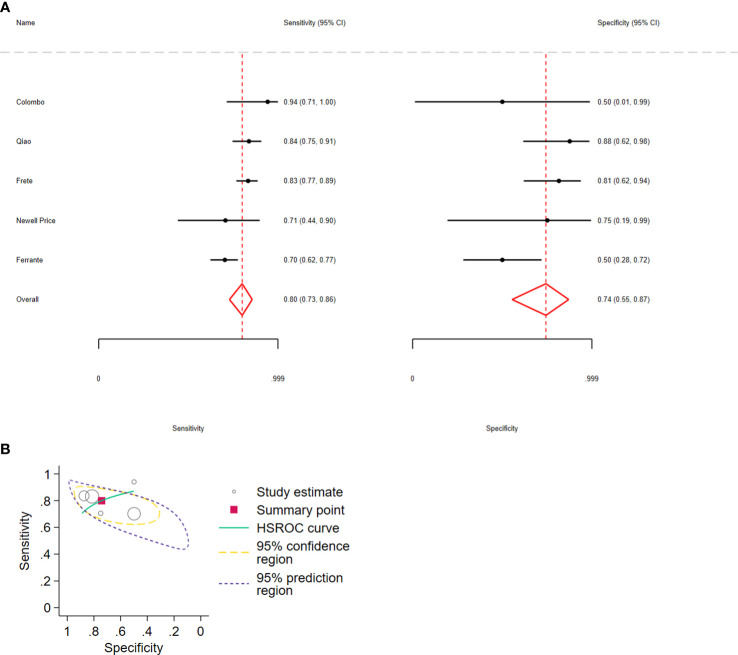
**(A)** Forest plot depicting the sensitivity and specificity considering the ACTH and cortisol percent increments after the 10 µg desmopressin test to distinguish Cushing disease from ectopic ACTH syndrome. Estimated study sensitivity and specificity (black circle); 95% confidence interval (black horizontal line). **(B)** Summary ROC plots from Stata after fitting the hierarchical model to ACTH and cortisol percent increment. Circles represent the estimates of individual primary studies, and squares indicate the summary points of sensitivity and specificity. HSROC curve is plotted as a curvilinear line passing through the summary point. The 95% confidence interval and 95% prediction interval are also provided. HSROC, hierarchical summary receiver operating characteristic.

Regarding the combination of ΔACTH > 35% and Δcortisol > 20%, the pooled sensitivity and specificity were 0.80 (95% CI: 0.73–0.86, I2 = 35%) and 0.74 (95% CI: 0.55–0.87, I2 = 27%), respectively; 5 studies, 511 participants, low certainty of evidence (**Figure 5A**; **Table 3**). The LR+ was 3 (95% CI: 1.58–67) and LR− was 0.23 (95% CI: 0.17–0.43).

In all analyses, compared with sensitivity, forest plots revealed greater variability in the estimated specificity across all studies. In addition, based on the graphical outputs obtained after fitting the hierarchical model, the 95% CIs were extremely wide, and the prediction intervals were wider than the CIs (**Figures 3B**, **4B**, **5B**).

#### 3.4.2 Distinguishing CD from NNH using the 10 μg desmopressin test

**Figure 6:**
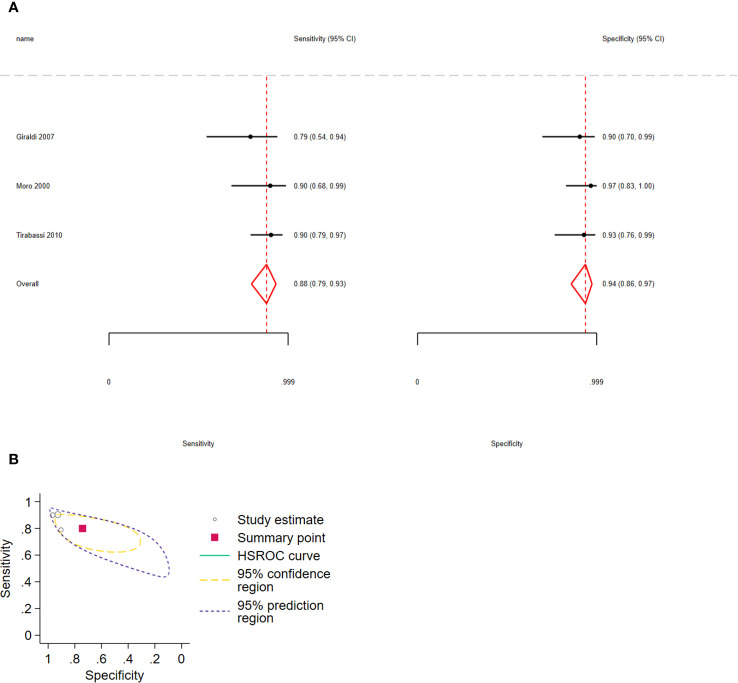
**(A)** Forest plot depicting the sensitivity and specificity considering the ACTH percent increment after the 10 µg desmopressin test to distinguish Cushing disease from non-neoplastic hypercortisolism. Estimated study sensitivity and specificity (black circle); 95% confidence interval (black horizontal line). **(B)** Summary ROC plots from Stata after fitting the hierarchical model to ACTH percent increment. Circles represent the estimates of individual primary studies, and squares indicate the summary points of sensitivity and specificity. HSROC curve is plotted as a curvilinear line passing through the summary point. The 95% confidence interval and 95% prediction interval are also provided. HSROC, hierarchical summary receiver operating characteristic.

The pooled sensitivity for distinguishing CD from NNH was 0.88 (95% CI: 0.79–0.93) and the specificity was 0.94 (95% CI: 0.86–0.97), 3 studies, 170 participants, very low certainty of evidence (**Figure 6**).

### 3.5 Quality of evidence

The quality of evidence regarding the desmopressin test for evaluating CD versus EAS was downgraded in two levels because of the risk of bias and uncertainty (all studies were evaluated as having an unclear risk of bias for patient selection, and prediction intervals in all pooled analyses were considerably wider than CIs). To evaluate CD versus NNH, the evidence was downgraded in three levels because of the risk of bias, uncertainty, and imprecision (a few participants per study). Publication bias could not be investigated because of the small number of studies included per meta-analysis (<10).

## 4 Discussion

Considering the need to differentiate CD from EAS and NNH, we evaluated the accuracy of the desmopressin test in these two clinical scenarios. We conducted a systematic literature review and found 14 studies that met our eligibility criteria. Based on the studies included in this review, 84 of 100 patients with ACTH-dependent syndrome will have CD (362/429) and 16 will have EAS (67/429). Of the 84 patients with CD, 13 (15%) will be misdiagnosed as not having CD based on the desmopressin test. Of the 16 patients with EAS, 6 (36%) will be falsely considered as having CD. The patients with EAS falsely diagnosed as having CD may have to undergo MRI. In the absence of an adenoma with a size of >6 mm, BIPSS will be performed, and the diagnosis may be rectified. Conversely, the patients with CD falsely diagnosed as having EAS would have to undergo an extensive investigation to determine the presence of ectopic ACTH production. Conversely, for patients with mild hypercortisolism, 48 and 52 of the 100 patients will have respectively CD and NNH. Among 48 patients with CD, the desmopressin test may misdiagnose 5 (11%) patients; however, these patients can be re-tested. Of the 52 patients without NHH, 4 may be unnecessarily referred for MRI and occasionally for BIPSS.

Although separate meta-analyses of each summary point seem to be extremely accurate in distinguishing CD from EAS and NNH, we revealed that the specificity decreased when sensitivity increased in all analyses. This occurred because separate pooling overlooks the correlation between sensitivity and specificity (20). The results of the separate meta-analyses of sensitivity and specificity are valid when the same criteria have been used for a positive result in each study, and each study is of similar size and quality (30). If different criteria or thresholds have been used, a relationship exists between sensitivity and specificity across all studies. This is known as the threshold effect (30). In these cases, weighted averages do not reflect the overall accuracy of the test (65). Therefore, the methods recommended for summarizing sensitivity and specificity are hierarchical and bivariate models. The bivariate model is preferred for computing summary points, whereas the HSROC model is preferred for constructing the HSROC curve (20). Additionally, the bivariate method focuses on determining the summary estimates of sensitivity and specificity and how these values vary with study‐level covariates. In contrast, the HSROC approach focuses on evaluating the SROC curve as the basis for assessing the accuracy of the test and investigating how the position and shape of the curve may vary with study‐level covariates (30). The confidence interval is related to the joint summary estimates of sensitivity and specificity in the HSROC space; however, this region does not represent the between-study heterogeneity (66). Conversely, the prediction interval refers to the sensitivity and specificity values that might be observed in a future study by describing the full extent of the uncertainty of summary points, which can thus reflect the between-study heterogeneity. In the current review, the 95% prediction intervals of all calculated HSROCs were wider than the 95% confidence intervals. Therefore, the certainty of evidence regarding the accuracy of the desmopressin test to distinguish CD from EAS and NHH was low/very low. This indicated that we have very little confidence of the estimated accuracy, and the true accuracy is likely to be significantly different from the estimated result.

Dynamic tests other than the desmopressin test have been used to distinguish CD from EAS (7). Corticotropic pituitary tumors are sensitive to CRH stimulation, whereas ectopic secretory tumors of ACTH are usually not sensitive to the stimulation (65–67). Therefore, the CRH test is employed based on this purpose (67, 68). However, considering its cost and unavailability in Brazil and worldwide, the use of the CRH test has been decreasing (7).

Furthermore, the HDDST has been used to distinguish the abovementioned two diagnoses (44, 69, 70). Although HDDST is associated with low cost and is readily available, its diagnostic accuracy is low, with 5%–25% of patients with EAS exhibiting suppression (4, 11, 47, 71) and up to 20% of patients with CD not exhibiting suppression (15). The previously adopted value for the suppression of serum cortisol levels (collected between 8 am and 9 am after fasting following ingestion of 8 mg dexamethasone at night) was 50% in patients with CD (23, 72–74). To improve the specificity of the test, some authors have proposed suppression of 80% of cortisol levels as the cutoff value (64, 70). However, this may result in a low level of accuracy (64).

A systematic review evaluating the diagnostic accuracy of the CRH test, desmopressin test, and HDDST for establishing a CD or EAS diagnosis revealed that the CRH test had the highest sensitivity for detecting CD on the basis of ΔACTH (87%) and Δcortisol (86%), along with the highest specificity for detecting EAS on the basis of ΔACTH (94%) and Δcortisol (89%). However, I2 values suggested substantial heterogeneity for sensitivity (62% ACTH and 78% cortisol), and no HSROCs were calculated (17).

The Dexa-CRH test (a test combining CRH administration after 48 h with 2 mg/day low-dose dexamethasone suppression test) has been previously used to distinguish CS from NNH (49). Yanovski first used the Dexa-CRH test to detect CS and proposed that a serum cortisol level of >1.4 μg/dL (absolute value) observed 15 min after the test is suggestive of CS (33). Erickson et al. (16) and Giraldi et al. (53) used this test to distinguish CD from NNH; based on the abovementioned proposed cortisol cutoff, they achieved a sensitivity of 100% and specificities of 76% and 62.5%, respectively. Erickson et al. (16) reported 95% sensitivity and 97% specificity in the ROC analysis of ACTH values of >27 pg/mL (5.9 pmol/L) at 15 min after CRH stimulus.

The most crucial limitation of this review was the number of studies included and the number of patients included per study (<100 in most studies) (75). When the number of studies is small, deciding which terms should be included in a model and which is the best model may be difficult. For both bivariate and HSROC models, estimates of variances of the random effects can be subject to a high level of uncertainty (30). Additionally, because a low number of studies were included per meta-analysis (<10), the presence of publication bias could not be evaluated. Moreover, we could not evaluate the sources of heterogeneity through subgroup analyses or meta-regression. Furthermore, the evaluated outcomes were limited by the diagnostic accuracy, and evaluation of other crucial aspects from the patient’s viewpoint, such as quality of life, stress, and costs incurred due to a false-positive diagnosis, was lacking.

Although we did not specify remission of hypercortisolism as a criterion for pituitary or ectopic ACTH overproduction, no study was excluded based on this, and we included studies in which CD was confirmed by remission of hypercortisolism after trans-sphenoidal surgery. Regarding the diagnostic approach to distinguish ACTH-dependent CS from ACTH-independent CS, persistent ACTH levels of >15 or >20 pg/mL have been used to diagnose ACTH-dependent hypercortisolism, ACTH levels of <5 or <10 pg/mL have been used to diagnose ACTH-independent hypercortisolism, and ACTH levels of 5–15 or 10–20 pg/mL have been reported as indeterminate, indicating that new samples should be ordered (7, 76). Indeterminate ACTH levels usually indicate ACTH-dependent cortisol secretion. Thus, to avoid losing studies that did not order new samples but instead used BIPSS and the presence of a pituitary adenoma measuring >6 mm on MRI to diagnose CD, we used a cutoff value of 10 pg/mL as an indication of ACTH-dependent hypercortisolism. Although some included studies did not use the ACTH value to distinguish these two diagnoses, all of them considered histopathological analyses, remission of hypercortisolism after pituitary surgery, or BIPSS results when diagnosing CD.

When this review was being performed, two other systematic reviews were published on the same topic. However, none of them summarized sensitivity and specificity using hierarchical and bivariate methods and presented certainty of evidence according to the GRADE approach (17, 77). Additionally, our review focused on the desmopressin test, an inexpensive and readily available test in most countries, which has been used as a substitute for the CRH test.

In conclusion, this evidence synthesis demonstrates that using the desmopressin test for distinguishing CD from EAS results in up to 20% of patients with CD being incorrectly diagnosed as EAS. Additionally, the use of the desmopressin test to distinguish CD from NNH results in 11% of patients with CD being falsely diagnosed as NNH and 7% of patients with NNH being falsely diagnosed as CD. Thus, the use of the desmopressin test alone is not recommended to distinguish CD from EAS or CD from NNH.

## Data availability statement

The original contributions presented in the study are included in the article/**Supplementary Material**. Further inquiries can be directed to the corresponding author.

## Author contributions

RG: Conceptualization, Data curation, Formal analysis, Investigation, Software, Writing – original draft. MC: Data curation, Formal analysis, Funding acquisition, Investigation, Methodology, Software, Writing – review & editing. EP: Data curation, Formal analysis, Investigation, Methodology, Software, Validation, Visualization, Writing – review & editing. MM: Conceptualization, Data curation, Investigation, Validation, Writing – review & editing. LV: Data curation, Investigation, Methodology, Validation, Writing – review & editing. VN-N: Conceptualization, Data curation, Formal analysis, Funding acquisition, Investigation, Methodology, Project administration, Resources, Software, Supervision, Validation, Visualization, Writing – original draft, Writing – review & editing.
