# Supplementary material for: Accuracy of the 10 μg desmopressin test for differential diagnosis of Cushing syndrome: a systematic review and meta-analysis
Source: Front Endocrinol (Lausanne). 2024 Jan 30;15:1332120. doi: 10.3389/fendo.2024.1332120 (PMC10861662; doi:10.3389/fendo.2024.1332120)
Supplement: Supplementary file 1 [file DataSheet_1.docx]

**SUPPLEMENTARY FILE**

**Repository: https://repositorio.unesp.br/server/api/core/bitstreams/9a5faa2d-5753-4e88-818b-ce16bb181c3f/content**

**Figure 1s**: Pooled ROC plots and its confidence interval from Stata, fitting the bivariate random-effects meta-analysis model to cortisol percent increment. Circles represent the estimates of individual primary studies, and square indicates the summary points of sensitivity and specificity. Area under the ROC curve (AUC), prediction counter and confidence counter are also provided.

**Figure 2s:** Pooled ROC plots and its confidence interval from Stata, fitting the bivariate random-effects meta-analysis model to ACTH percent increment. Circles represent the estimates of individual primary studies, and square indicates the summary points of sensitivity and specificity. Area under the ROC curve (AUC), prediction counter and confidence counter are also provided.

**Search Strategies**

**01/10/2019**

**Updated 25/09/2023**

**PUBMED**

#1 "**Pituitary ACTH Hypersecretion"[Mesh]** OR (ACTH Hypersecretion, Pituitary) OR (Hypersecretion, Pituitary ACTH) OR (Cushing Disease, Pituitary) OR (Cushing Diseases, Pituitary) OR (Pituitary Cushing Disease) OR (Pituitary Cushing Diseases) OR (Pituitary-Dependant Hypercortisolism Disorder) OR (Hypercortisolism Disorder, Pituitary-Dependant) OR (Hypercortisolism Disorders, Pituitary-Dependant) OR (Pituitary Dependant Hypercortisolism Disorder) OR (Pituitary-Dependant Hypercortisolism Disorders) OR (Pituitary-Dependant Cushing Syndrome) OR (Cushing Syndrome, Pituitary-Dependant) OR (Pituitary Dependant Cushing Syndrome) OR (Pituitary-Dependant Hypercortisolism) OR (Hypercortisolism, Pituitary-Dependant) OR (Hypercortisolisms, Pituitary-Dependant) OR (Pituitary Dependant Hypercortisolism) OR (Pituitary-Dependant Hypercortisolisms) OR (Cushing Disease) OR (Disease, Cushing) OR (Pituitary Cushing Syndrome) OR (Cushing Syndrome, Pituitary) OR (Inappropriate ACTH Secretion Syndrome) OR (Inappropriate Adrenocorticotropic Hormone Secretion) OR (Adrenocorticotropic Hormone, Inappropriate Secretion) OR "**Cushing Syndrome"[Mesh]** OR (Syndrome, Cushing) OR (Cushing's Syndrome) OR (Syndrome, Cushing's) OR (Hypercortisolism) OR (ACTH Secreting Pituitary Adenoma) OR "**ACTH-Secreting Pituitary Adenoma"[Mesh]** OR (ACTH-Secreting Pituitary Adenomas) OR (Pituitary Adenomas, ACTH-Secreting) OR (Corticotroph Adenoma) OR (Adenoma, Corticotroph) OR (Adenomas, Corticotroph) OR (Corticotroph Adenomas) OR (Pituitary Corticotropin-Secreting Adenoma) OR (Corticotropin-Secreting Adenoma, Pituitary) OR (Corticotropin-Secreting Adenomas, Pituitary) OR (Pituitary Corticotropin Secreting Adenoma) OR (Pituitary Corticotropin-Secreting Adenomas) OR (ACTH-Producing Pituitary Adenoma) OR (ACTH Producing Pituitary Adenoma) OR (ACTH-Producing Pituitary Adenomas) OR (Pituitary Adenoma, ACTH-Producing) OR (Pituitary Adenomas, ACTH-Producing) OR (Pituitary Adenoma, ACTH-Secreting) OR (Pituitary Adenoma, ACTH Secreting)

#2 **"Deamino Arginine Vasopressin"[Mesh]** OR (Vasopressin, Deamino Arginine) OR (Arginine Vasopressin, Deamino) OR (Desmopressin) OR (1-Deamino-8-D-arginine Vasopressin) OR (Vasopressin, 1-Deamino-8-D-arginine) OR (1-Desamino-8-arginine Vasopressin) OR (Vasopressin, 1-Desamino-8-arginine) OR (Adiuretin SD) OR (Apo-Desmopressin) OR (Apotex Brand of Deamino Arginine Vasopressin) OR (DDAVP) OR (Desmospray) OR (Octostim) OR (Ferring Brand 1 of Desmopressin Acetate) OR (Octim) OR (Desmopressine Ferring) OR (Ferring, Desmopressine) OR (Desmotabs) OR (Nocutil) OR (Norgine Brand of Desmopressin Acetate) OR (Hoyer Brand of Desmopressin Acetate) OR (Desmopressin Acetate) OR (Acetate, Desmopressin) OR (Desmopressin Monoacetate, Trihydrate) OR (Monoacetate, Trihydrate Desmopressin) OR (Trihydrate Desmopressin Monoacetate) OR (Desmopressin Monoacetate) OR (Monoacetate, Desmopressin) OR (IQFA Brand of Desmopressin Acetate) OR (Minirin) OR (Minurin) OR (Ferring Brand 2 of Desmopressin Acetate) OR (Adiuretin) OR (Desmogalen) OR (Galen Brand of Desmopressin Acetate) OR (Ddavp test* ) OR **"Corticotropin-Releasing Hormone"[Mesh]** OR (Corticotropin Releasing Hormone) OR (ACTH-Releasing Hormone) OR (ACTH Releasing Hormone) OR (Corticoliberin) OR (CRF-41) OR (Corticotropin-Releasing Factor-41) OR (Corticotropin Releasing Factor 41) OR (Corticotropin-Releasing Hormone-41) OR (Corticotropin Releasing Hormone 41) OR (CRF (ACTH)) OR (ACTH-Releasing Factor) OR (ACTH Releasing Factor) OR (Corticotropin-Releasing Factor) OR (Corticotropin Releasing Factor)

**Total: 1268**

**EMBASE**

**'Cushing disease'**/exp OR ‘adrenocortical hyperplasia, acth induced’ OR ‘corticotropin induced adrenocortical hyperplasia’ OR ‘Cushing disease’ OR ‘cushing syndrome, acth induced’ OR ‘Cushings disease’ OR ‘itsenko cushing disease’ OR ‘pituitary ACTH hypersecretion’ OR **'ACTH secreting adenoma'**/exp OR ‘ACTH-secreting pituitary adenoma’ OR ‘ACTH producing adenoma’ OR ‘ACTH producing adenomas’ OR ‘ACTH producing pituitary adenoma’ OR ‘ACTH producing pituitary adenomas’ OR ‘ACTH producing pituitary tumor’ OR ‘ACTH producing pituitary tumors’ OR ‘ACTH producing pituitary tumour’ OR ‘ACTH producing pituitary tumours’ OR ‘ACTH producing tumor’ OR ‘ACTH producing tumors’ OR ‘ACTH producing tumour’ OR ‘ACTH producing tumours’ OR ‘ACTH secreting adenomas’ OR ‘ACTH secreting pituitary adenoma’ OR ‘ACTH secreting pituitary adenomas’ OR ‘ACTH secreting pituitary tumor’ OR ‘ACTH secreting pituitary tumors’ OR ‘ACTH secreting pituitary tumour’ OR ‘ACTH secreting pituitary tumours’ OR ‘ACTH secreting tumor’ OR ‘ACTH secreting tumors’ OR ‘ACTH secreting tumour’ OR ‘ACTH secreting tumours’ OR ‘adrenocorticotropic hormone secreting adenoma’ OR ‘corticotroph adenoma’ OR ‘corticotroph adenomas’ OR ‘corticotropinoma’ OR ‘corticotropinomas’

**'desmopressin'/exp** OR ‘(1 deamino 8 dextro arginine) vasopressin’ OR ‘1 deamine 8 d arginine vasopressin’ OR ‘1 deamino 8 d arginine vasopressin’ OR ‘1 deamino 8 dextro arginine vasopressin’ OR ‘1 desamino 8 d arginine vasopressin’ OR ‘8 (1 desaminoarginine) vasopressin’ OR ‘[1 deamino 8 d arginine] vasopressin’ OR ‘[1 deamino 8 dextro arginine] vasopressin’ OR ‘[deamino 8 cysteine d arginine] vasopressin’ OR ‘[deamino 8 cysteine dextro arginine] vasopressin’ OR ‘[deamino 8 dextro arginine] vasopressin’ OR ‘adin’ OR ‘adiuretin’ OR ‘adiuretin sd’ OR ‘adiuretin-sd’ OR ‘concentraid’ OR ‘d-void’ OR ‘dav ritter’ OR ‘DDAVP’ OR ’DDAVP (needs no refrigeration)’ OR ‘DDAVP desmopressin’ OR ‘DDAVP melt’ OR ‘DDAVP nasal’ OR ‘DDAVP rhinal tube’ OR ‘DDAVP tablets’ OR ‘deamino 8 dextro arginine vasopressin’ OR ‘deamino 8d arginine vasopressin’ OR ‘deamino dextro arginine vasopressin’ OR ‘deaminovasopressin [8 d arginine]’ OR ‘defirin' OR 'defirin melt’ OR ‘desmirin’ OR ‘desmomelt’ OR ‘desmopresina’ OR ‘desmopressin acetate’ OR 'desmopressin acetate (needs no refrigeration)’ OR ‘desmopressin acetate preservative free’ OR ‘desmopressin diacetate’ OR ‘desmopressin nasal solution’ OR ‘desmopressina’ OR ‘desmopressine’ OR ‘desmospray' OR ‘desmotab’ OR ‘desmotabs’ OR ‘desmotabs melt’ OR ‘desurin’ OR ‘emosint’ OR ‘enupresol’ OR ‘minirin’ OR ‘minirin DDAVP’ OR ‘minirin melt’ OR ‘minirin nasal spray’ OR ‘minirin rhinetten’ OR ‘minirin rhinyle’ OR ‘minirin spray’ OR ‘minirinette’ OR ‘minirinmelt’ OR ‘minrin’ OR ‘minurin’ OR ‘minurin flas’ OR ‘minurin gotas’ OR ‘miram’ OR ‘nictur’ OR ‘noctisson’ OR ‘nocturin’ OR ‘nocutil’ OR ‘nordurine’ OR ‘novidin’ OR ‘nucotil nasenspray’ OR ‘octim’ OR ‘octostim’ OR ‘octostim nasal spray’ OR ‘octostim spray’ OR ‘presinex’ OR ‘stimate’ OR ‘vasopressin 8 (1 desaminoarginine)’ OR ‘vasopressin’ OR ‘1 deamino 8 dextro arginine’ OR ‘vasopressin’ OR ‘deamino 8 dextro arginine’ OR ‘vasopressin [1 (3 mercaptopropionic acid) 8 dextro arginine]’ OR ‘vasopressin [1 deamino 8 dextro arginine]’ OR ‘vasopressin [deamino 8 cysteine dextro arginine]’ OR ‘vasopressin [deamino 8 d arginine]’ OR ‘vasopressin [deamino 8 dextro arginine]’ OR ‘vasopressin [deamino dextro arginine]' OR 'wetirin’

**Total: 449**

**LILACS / IBECS/ MEDCARIB / BBO / BDENF (BVS – IAHX)**

- Descriptor in portuguese: Hipersecreção Hipofisária de ACTH
- Synonyms in portuguese:
- Hipersecreção de ACTH Hipofisária
- Hipersecreção de ACTH Pituitária
- Hipersecreção Pituitária de ACTH
- Doença de Cushing
- Síndrome da Secreção Inadequada de ACTH
- Secreção Inadequada de Hormônio Adrenocorticotrópico
- C10.228.140.617.738.250.725
- C19.700.355.800
- Descriptor in portuguese: Síndrome de Cushing
- C19.053.800.367
- Descriptor in portuguese: Desamino Arginina Vasopressina
- Synonyms in portuguese:
- Desmopressina
- Desaminoarginina Vasopressina
- D06.472.699.631.692.781.100.250
- D12.644.400.900.100.250
- D12.644.456.925.100.250
- D12.644.548.691.692.781.100.250
- D12.776.641.650.937.100.250
- Search strategy
- #1 MH:"Hipersecreção Hipofisária de ACTH" OR (Hipersecreção de ACTH Hipofisária) OR
- (Hipersecreção de ACTH Pituitária) OR (Hipersecreção Pituitária de ACTH) OR (Doença de Cushing) OR
- (Síndrome da Secreção Inadequada de ACTH) OR (Secreção Inadequada de Hormônio Adrenocorticotrópico)
- OR MH:C10.228.140.617.738.250.725$ OR MH:C19.700.355.800$
- #2 MH:"Síndrome de Cushing" OR
- MH:C19.053.800.367$
- #3 MH:"Desamino Arginina Vasopressina" OR (Desmopressina) OR (Desaminoarginina Vasopressina)
- OR MH: D06.472.699.631.692.781.100.250$ OR MH: D12.644.400.900.100.250$ OR MH: D12.644.456.925.100.250$
- OR MH: D12.644.548.691.692.781.100.250$ OR MH: D12.776.641.650.937.100.250$

**Total: 55**

**CENTRAL - COCHRANE**

ID Search Hits

#1 MeSH descriptor: [Pituitary ACTH Hypersecretion] explode all trees 18

#2 MeSH descriptor: [Cushing Syndrome] explode all trees 88

#3 MeSH descriptor: [Deamino Arginine Vasopressin] explode all trees 345

#4 Cushing disease 312

#5 #1 or #2 or #4 352

#6 #3 and #5 7

**Total: 7**
